# Enhanced electrocatalytic activity of fluorine doped tin oxide (FTO) by trimetallic spinel ZnMnFeO_4_/CoMnFeO_4_ nanoparticles as a hydrazine electrochemical sensor

**DOI:** 10.1038/s41598-023-39321-0

**Published:** 2023-07-27

**Authors:** Jalal Niazi Saei, Karim Asadpour-Zeynali

**Affiliations:** 1 Department of Analytical Chemistry, Faculty of Chemistry, University of Tabriz, Tabriz, 51666-16471 Iran; 2 Pharmaceutical Analysis Research Center, Faculty of Pharmacy, Tabriz University of Medical Sciences, Tabriz, 51664 Iran

**Keywords:** Analytical chemistry, Catalysis, Electrochemistry, Electrocatalysis

## Abstract

In the present study, ZnMnFeO_4_ and CoMnFeO_4_ tri-metallic spinel oxide nanoparticles (NPs) were provided using hydrothermal methods. The nanoparticles have been characterized by X-ray diffraction (XRD), field emission scanning electron microscopy (FESEM), Fourier transform infrared spectroscopy (FTIR), energy-dispersive X-ray spectroscopy (EDX), transmission electron microscopy (TEM), and electrochemical techniques. A reliable and reproducible electrochemical sensor based on ZnMnFeO_4_/CoMnFeO_4_/FTO was fabricated for rapid detection and highly sensitive determination of hydrazine by the DPV technique. It is observed that the modified electrode causes a sharp growth in the oxidation peak current and a decrease in the potential for oxidation, contrary to the bare electrode. The cyclic voltammetry technique showed that there is high electrocatalytic activity and excellent sensitivity in the suggested sensor for hydrazine oxidation. Under optimal experimental conditions, the DPV method was used for constructing the calibration curve, and a linear range of 1.23 × 10^−6^ M to 1.8 × 10^−4^ M with a limit of detection of 0.82 ± 0.09 μM was obtained. The obtained results indicate that ZnMnFeO_4_/CoMnFeO_4_/FTO nano sensors exhibit pleasant stability, reproducibility, and repeatability in hydrazine measurements. In addition, the suggested sensor was efficiently employed to ascertain the hydrazine in diverse samples of cigarette tobacco.

## Introduction

The application of metal oxide nanoparticles has recently grown significantly in photocatalytic and sensor applications^1^. Besides, given the high catalytic action, inexpensiveness, and chemical stability of these materials, many applications of them have been developed in energy^2^. Transition metal oxide nanoparticles also show great photocatalytic and electrical properties due to their shape, size, and area^3,4^. Spinel oxides are such materials that contain one or more transition metals in their structure, such as Fe_3_O_4_^5^ and MgFe_2_O_4_^6^, which are used as electrodes in rechargeable supercapacitors and batteries^7,8^.

A recent discovery has indicated that trimetallic spinel oxides exhibit enhanced properties in comparison to their monometallic and bimetallic counterparts when employed as electrode materials in lithium-ion batteries. Lavela and colleagues synthesized NiFeMnO_4_ utilizing a reverse micelle technique and achieved a substantial capacity of approximately 900 mAh/g, as reported in their study^9^. Stefan et al. synthesized the CoMnFeO_4_ nanoparticles and reported their superior electrochemical performance compared to several other binary oxides^10^. Based on a fundamental principle or set of principles, the following statement is made: the trimetallic oxides comprising Co, Fe, and Mn metals have been identified as a potentially effective catalyst for the development of a high-performing Advanced Oxidation Process (AOP) system, as stated previously. Conversely, the inclusion of the Fe oxide constituent will confer exceptional magnetic characteristics upon the catalyst, thereby facilitating its recyclability^11^. Among these structures, trimetallic spinel oxides, such as CoMnFe_2_O_4_, have been neglected, despite the fact that they are indeed likely to have a simple synthesis and morphology^12^, and given that the response of electrochemical sensors has a major dependence on the morphology and size of electrocatalyst particles and the effective area of the modified electrode, these materials can be considered intriguing and efficient catalysts^13^.

In this study, CoMnFeO_4_ and ZnMnFeO_4_ NPs, as modifiers for measuring hydrazine, were synthesized via the hydrothermal technique and deposited on the FTO glass. The ionic configuration of CoMnFeO_4_ is analogous to that of CoMnFe_2_O_4_, wherein (Fe^3+^Co^2+^) [Fe^3+^Mn^3+^Mn^4+^Co^2+^] O_4_^2−^ is present. The parentheses and brackets indicate the tetrahedral (A site) and octahedral (B site), respectively, while O represents oxygen. This information has been reported in Ref.^12^:

$$\left( {{\text{Fe}}^{3 + } {\text{Mn}}^{2 + } {\text{Co}}^{2 + } } \right) \, \left[ {{\text{Fe}}^{3 + } {\text{Mn}}^{3 + } {\text{Mn}}^{4 + } {\text{Co}}^{2 + } } \right]{\text{ O}}_{4}^{2 - }$$

In addition, it has been observed that under different preparation conditions, such as changes in temperature, the stability of Fe^2+^, Mn^2+^ and Mn^3+^ ions are compromised, leading to their oxidation in the temperature range of 200–450 °C. This ultimately results in the formation of defective spinel ferrite. Furthermore, it has been observed that Mn^4+^ ions undergo reduction to Mn^3+^ ions at temperatures exceeding 450 °C^14^. The ionic configuration of ZnMnFeO_4_ exhibits a resemblance to that of CoMnFeO_4_, which was explained.

Hydrazine is an inorganic compound with the molecular formula N_2_H_4_ and is also a colorless liquid with an ammonia-like odor^15^. It is one of the environmental pollutants and a carcinogenic compound that can enter the body through the skin, lungs, and digestive system^16,17^. Furthermore, hydrazine is known as the stimulus of the nervous system, the gene mutation agent, and the cause of hematic abnormalities, and it has harmful side effects on the brain^1,18^. Consequently, the United States Environmental Protection Agency (EPA) has implemented a threshold level of 10 ppb for hydrazine^19^. However, hydrazine has a variety of applications in different industries, despite its toxicity. For example, it is used as fuel for spacecraft and missiles due to its rapid combustion reactions. Moreover, it is widely used to remove oxygen from steam boilers and to prevent the corrosion of boiler tubes in power plants^1,20^. More importantly, hydrazine is useful in the agricultural industry, in which tobacco products as well as insecticides are produced^21^. This compound is also a strong reducing agent^17,22^. Therefore, the measurement of hydrazine is important in terms of its being widely used and its toxicity, which has led to the appearance of a lot of methods to measure it, including potentiometric and titration^23,24^, spectrophotometry^25^, chromatography^26^, fluorimetry^27^, chemiluminescence^28^ and colorimetry^29^. According to the ability of hydrazine for electrochemical oxidation, electrochemical methods are also used as efficacious methods to measure it^30^.

Among the advantages of this method, the inexpensiveness, short response time, quick and easy sample preparation, high selectivity and sensitivity, and portability can be explicitly cited^20,31,32^. Despite the great benefits of this method, the measurement of hydrazine on unmodified electrodes has low sensitivity and great disturbance. By modifying these electrodes with proper materials that can catalyze the oxidation of hydrazine, the hydrazine oxidation over voltage can be diminished, followed by increasing the sensitivity of hydrazine and decreasing the disturbance in its measurement. A proper modifier can increase the efficient surface area of the electrode and the electron transfer velocity and can enhance and reinforce electrochemical reactions as a catalyst. In recent years, the use of nanomaterials with high electrocatalytic stability, such as metal and metal oxide nanoparticles^33^, conducting polymers^34^, carbon nano-tubes^35^ or graphene^36^, has been introduced for electrochemical measurement of hydrazine.

**Scheme 1:**
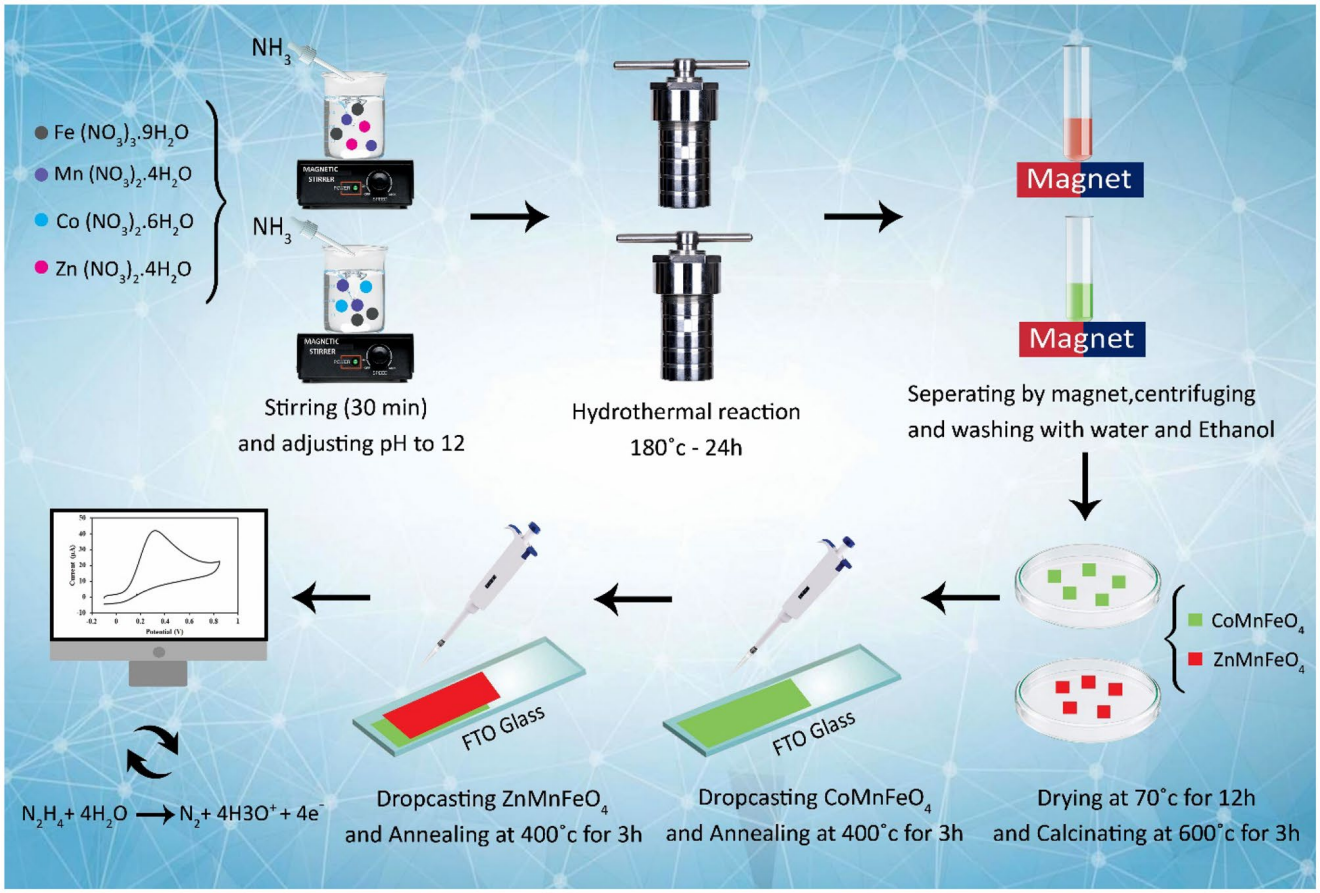
Schematic representation of hydrazine detection using ZnMnFeO_4_/CoMnFeO_4_/FTO.

To measure hydrazine simply, sensitively, accurately, and cheaply, CoMnFeO_4_ and ZnMnFeO_4_ were used as modifiers to prepare an electrochemical sensor of hydrazine (Scheme 1). Afterward, the behavior of hydrazine on the modified FTO electrode was electrochemically examined by as-synthesized NPs and a bare FTO electrode. One notable benefit of utilizing FTO as a substrate in this study is the absence of a binder in the nanoparticle deposition process. The nanoparticles were deposited and stabilized on the electrode surface through heating. A temperature of 400° was deemed necessary for the completion of this task. Hence, it is not feasible to employ alternative substrates such as glassy carbon, nickel foam, copper foam, etc., which are unsuitable for employment in this context. One significant limitation of this substrate is its limited reusability, as it is not feasible for utilization beyond three repetitions.

According to the literature, the trimetallic spinel oxide NPs have been used for the first time as a modifier to determine hydrazine, and this is also the first time that the electrocatalytic behavior of ZnMnFeO_4_ nanoparticles has been investigated. This modified electrode is used to determine the amount of hydrazine in diverse tobacco samples.

## Experimental

The apparatus, reagents, and solutions used in this paper are reported in the Electronic Supplementary Material File.

### Hydrothermal synthesis of CoMnFeO_4_ and ZnMnFeO_4_ nanoparticles

The process of hydrothermal synthesis of ZnMnFeO_4_ nanoparticles, which was carried out significantly in this study, was conducted similarly to the synthesis process of CoMnFeO_4_^12,37^. In 50 mL of distilled water, a particular amount of Mn(NO_3_)_2_·4H_2_O (2 mM), Fe(NO_3_)_3_·9H_2_O (4 mM), and Zn(NO_3_)_2_·6H_2_O (6 mM) were dissolved and stirred by a magnetic stirrer for 30 min to obtain a homogenous solution. Following that, the pH was adjusted to 12.0 by adding the ammonia aqueous solution gradually and dropwise to the solution under stirring, and then the cations' hydroxides were coprecipitated. Next, the obtained mixture was transmitted into a 100-mL Teflon™-lined autoclave and heated at 180 °C for 24 h. After the final product reached ambient temperature, it was separated by a magnet, centrifuged, and washed with distilled water and ethanol several times until a neutral pH was achieved. Then, the product was placed at 70° inside the oven for 12 h and dried completely. Thereafter, the as-synthesized powder was calcined at 600 °C for 3 h.

### Fabrication of modified electrode

In the first phase, it’s extremely important to prepare and activate the substrate surface before modifying the electrode. As a consequence, FTO glass was degreased and cleaned by ultrasonication for 30 min, which was implemented in 3 phases, including cleaning in distilled water, acetone, and ethanol, respectively. The time spent on each phase is 10 min. At last, it was allowed to be dried under a flow of nitrogen gas immediately before use. Then, 1.5 mg of synthesized CoMnFeO_4_ nanoparticles were dispersed in 1 mL of *N*-methyl pyrrolidone via an ultrasonic bath for 10 min. This suspension (10 µL volume) was drop-cast on a 1 cm × 1 cm FTO glass substrate and dried in an oven at 80 °C for 1 h. Next, it was stabilized and annealed in air at 400 °C for 3 h. In the end, a light tan film was obtained on the FTO substrates. Following this, the same phases were carried out to deposit ZnMnFeO_4_ on the CoMnFeO_4_ electrode. After all, the modified electrode, which is indicated as ZnMnFeO_4_/CoMnFeO_4_/FTO was rinsed with deionized water.

## Results and discussion

### Characterizations of the morphology and structure

**Figure 1:**
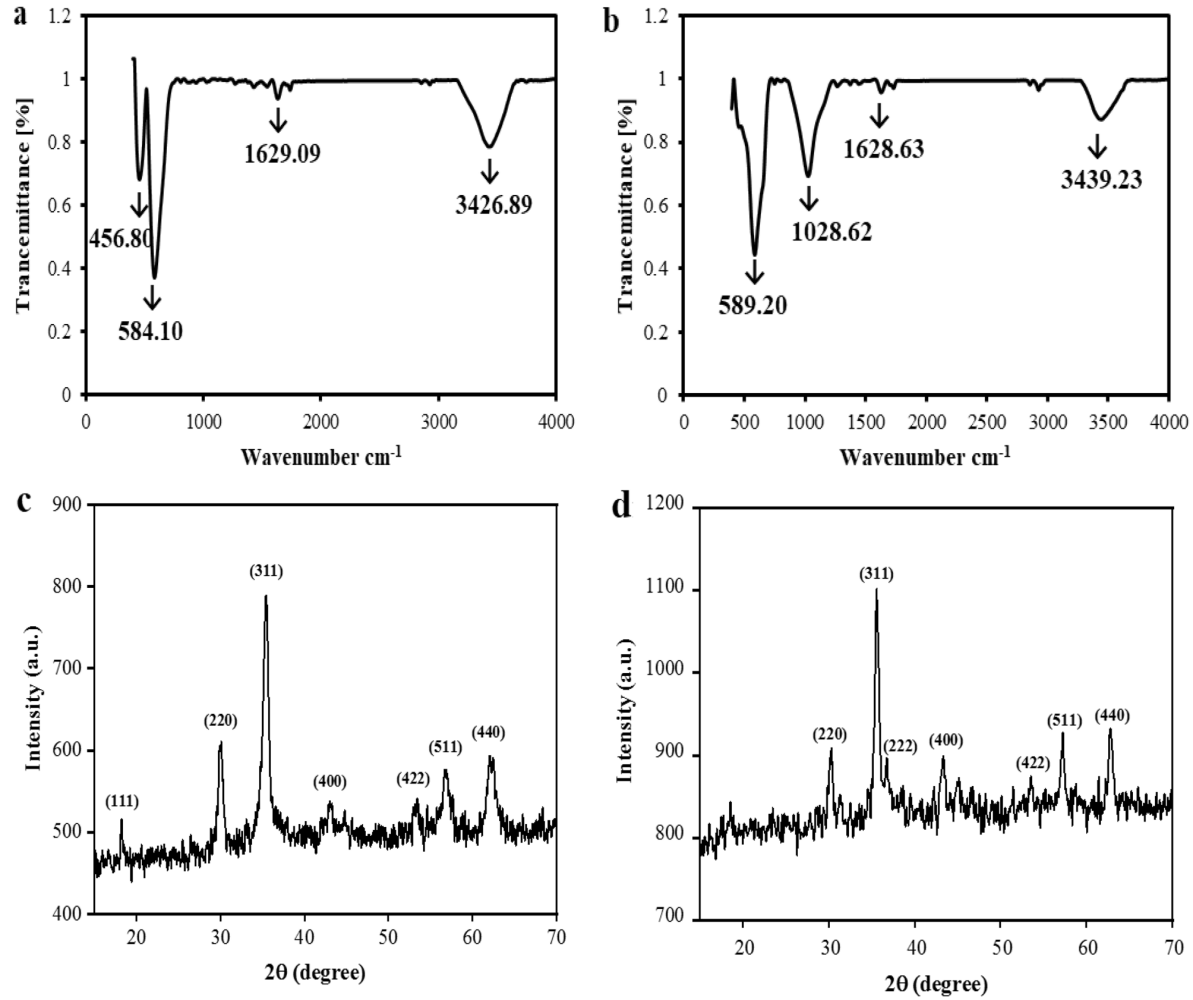
FT-IR spectrum and XRD pattern of synthesized (**a**,**c**) ZnMnFeO_4_ NPs and (**b**,**d**) CoMnFeO_4_ NPs.

The spinel ferrites are used in electrochemical sensors because of some features, including their superlative electrical and photoelectrochemical performance, high chemical stability, magnetic properties, low price, and good conductivity. Briefly, the modifier used in the present study causes HZ response enhancement and oxidation potential reduction considerably, which is due to its exceptional conductivity, high adsorption, and high electrochemical surface area of ZnMnFeO_4_/CoMnFeO_4_/FTO. By means of the FT-IR spectra presented in Fig. 1, the structural formation and functional groups that are provided in the as-synthesized samples, together with their metal-oxide vibrational modes, are acknowledged explicitly. In the IR spectra of all the ferrite samples, there are generally two metal-oxide bands observed, which both pertain to the nature of octahedral M–O stretching vibration and the nature of tetrahedral M–O stretching vibration^38^. In the spectrum of ZnMnFeO_4_ (Fig. 1a), the band with a greater wave number viewed at 584 cm^−1^ is ascribed to intrinsic stretching vibrations of the metal ions (Zn–O and Fe–O) at the tetrahedral site, while the other band viewed at 456 cm^−1^ correlates to the bending vibrations of the metal ions (Zn–O, Mn–O, and Fe–O) at the octahedral site^38^. In the FT-IR spectrum of CoMnFeO_4_ (Fig. 1b), the absorption band emerges at 589 cm^−1^ as a result of the intrinsic stretching vibrations of metal ions (Co–O and Fe–O) bonding the tetrahedral site^12^. The absorption peak that can be seen at 1630 and 3440 cm^−1^ corresponds to water molecules^39^. The CoMnFeO_4_ and ZnMnFeO_4_ ferrite samples are synthesized by the hydrothermal route, and in Fig. 1c,d, the patterns of X-ray diffraction for these samples can be clearly seen. For both samples, which have proper crystallinity and well-defined diffraction lines, a single-phase spinel structure was perceived without any unfavorable phase. There is no obvious peak detected for both samples. This bears witness to the high purity of the synthesized nanostructures. In Fig. 1c, the Apparent diffraction peaks for synthesized ZnMnFeO_4_ nanoparticles at planes of (111), (220), (311), (400), (422), (511), and (440) adapted well to the standard pattern reported in JCPDS card no. 01-074-2400. And in Fig. 1d, the relevant peaks of CoMnFeO_4_ at planes of (220), (311), (222), (400), (422), (511), and (440) in the XRD pattern could be indexed to the standard pattern reported in JCPDS card no. 00-001-1121. Based on the XRD diffractograms of both nanoparticles, it can be seen that every one of the peaks is either all even or all odd. This indicates that the samples are spinel in phase^40^.

**Figure 2:**
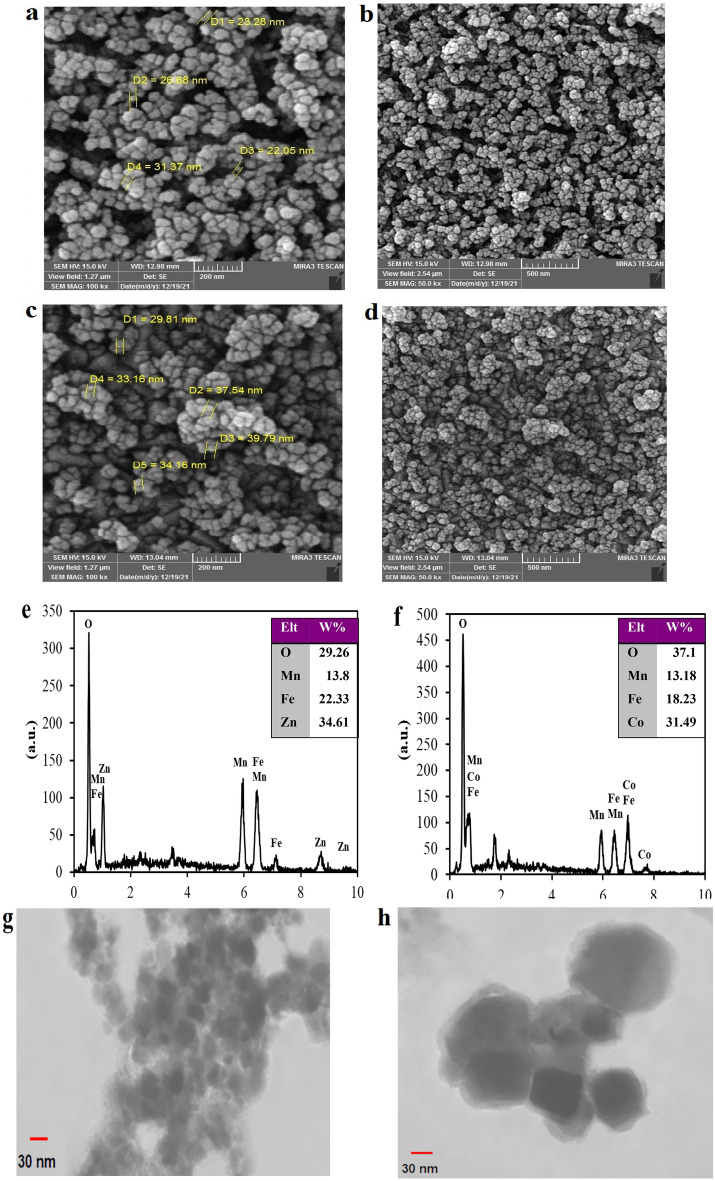
FE-SEM images and EDX spectra of synthesized (**a**,**b**,**e**) ZnMnFeO_4_ NPs and (**c**,**d**,**f**) CoMnFeO_4_ NPs with the scales of 200 nm and 500 nm; (**g**,**h**) TEM images of ZnMnFeO_4_ and CoMnFeO_4_ nanoparticles, respectively.

The better electrochemical sensor response is predominantly dependent on the electrode surface area, which was utilized in modified electrodes, and the size and morphology of the electrocatalysts. The purpose of the FE-SEM implementation was to investigate the morphological features and particle sizes of ZnMnFeO_4_ and CoMnFeO_4_ NPs. The synthesized ZnMnFeO_4_ sample’s SEM image is shown in Fig. 2a,b. In conformity with this delineation, the size of nanoparticles in these nanostructures tends to be smaller than CoMnFeO_4_, less than about 35 nm in diameter. The image of CoMnFeO4 nanoparticles less than 45 nm in size can be seen in Fig. 2c,d. According to the images, it can be stated that both synthesized nanoparticles resemble each other in some ways, including morphology, uniform size distribution, and compact arrangement. They can be illustrated as broccoli-bearing, multi-piece platelets that are connected to one object. The composition of the ZnMnFeO_4_ and CoMnFeO_4_ NPs electrode surface elements was designated with X-ray energy scattering spectroscopy. Both EDX spectra revealed a peak at 0.51 keV for O Kα. The revealed peak is because of the oxygen atoms in ZnMnFeO_4_ and CoMnFeO_4_ nanoparticles. There are three exclusive peaks for the Zn, Mn, and Fe elements in the ZnMnFeO_4_ graph (Fig. 2e) and also for the Co, Mn, and Fe elements in the CoMnFeO_4_ graph (Fig. 2f). The presence of desired elements was confirmed by the findings in the prepared compositions, which are uniformly distributed without the appearance of any impurities. Given that, it leads to the conclusion that the synthesis of ZnMnFeO_4_ and CoMnFeO_4_ has been efficiently accomplished. As shown in Fig. 2g,h, the morphologies of ZnMnFeO_4_ and CoMnFeO_4_ were examined by TEM. The stickled, truncated cubic particles were detectable in both images. The size of its particles for ZnMnFeO_4_ was less than 35 nm, and for CoMnFeO_4_, it was less than about 45 nm in diameter.

### Electrochemical characterization of the HZ sensor

**Figure 3:**
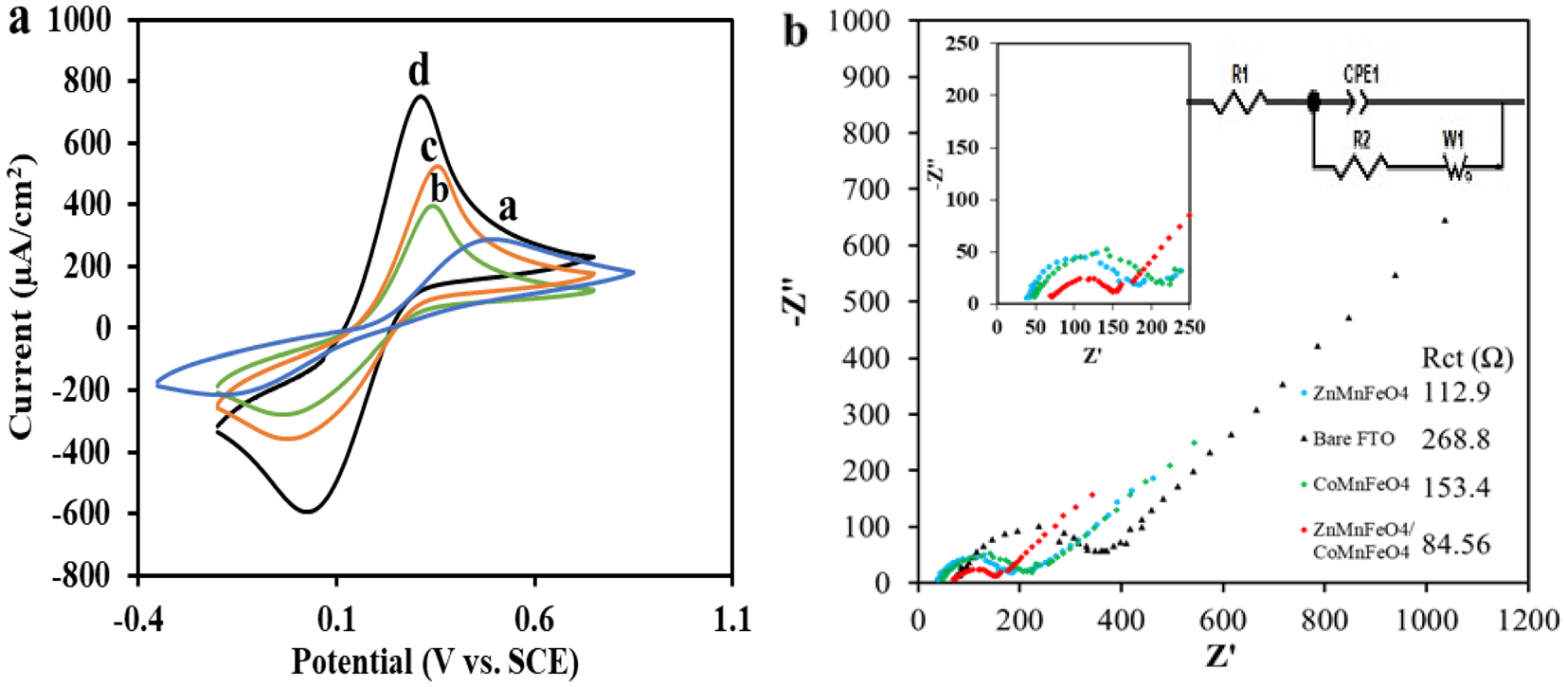
(**a**) Cyclic voltammograms for varied electrodes in 5mM [Fe(CN)_6_]^−3^/[Fe(CN)_6_]^−4^ with KCl 0.1 M as supporting electrolyte at the scan rate of 50 mV s^−1^. (a) Bare FTO; (b) CoMnFeO_4_/FTO; (c) ZnMnFeO_4_/FTO; (d) ZnMnFeO_4_/CoMnFeO_4_/FTO; (**b**) Electrochemical impedance spectroscopy (EIS) of Bare FTO and the other modified electrodes in 5 mM [Fe(CN)_6_]^−3^/[Fe(CN)_6_]^−4^ with KCl 0.1 M.

In order to modify the FTO substrate, the ZnMnFeO_4_/CoMnFeO_4_ NPs have been used as modifiers, and to control the tests, the FTO substrate was also modified with CoMnFeO_4_ and ZnMnFeO_4_ and then defined as CoMnFeO_4_/FTO and ZnMnFeO_4_/FTO. The electrochemical behavior of the three freshly modified electrodes, including ZnMnFeO_4_/CoMnFeO_4_/FTO, CoMnFeO_4_/FTO, and ZnMnFeO_4_/FTO, in 0.1 M KCl as a carrier electrolyte and in 5 mM [Fe(CN)_6_]^−3^/[Fe(CN)_6_]^−4^ as a redox probe at a scan rate of 50 mV s^−1^, were evaluated by using the cyclic voltammetry technique significantly and were compared to each other (Fig. 3a). According to the results, the modification of electrodes with ZnMnFeO_4_/CoMnFeO_4_ created a reduction in the peak-to-peak separation and had higher peak currents compared to the others. In fact, the features of the modified electrode, such as higher electrical conductivity and the large electroactive surface area, make these significant differences between the bare electrode and the ZnMnFeO_4_/CoMnFeO_4_/FTO electrode.

The electrical conductivity and electron transition resistance of the modified electrode surface have been checked by electrochemical impedance spectroscopy. The EIS spectrum record of the bare FTO electrode and the three modified electrodes cited above is shown in Fig. 3b. Studying the process of charge transfer on the surface of the modified electrode is a practical way to do so. The reason is that there is a double-layer capacitance and also a resistance to interfacial charge transfer after the modification on the electrode surface. The EIS Nyquist spectra involve a semicircular region and a linear region. The semicircular diameter in the high-frequency region indicates the resistance of the interface charge transfer (Rct) that is shown in Fig. 3b. The Warburg element represents the diffusion process and is associated with the low-frequency region linear section. The transition resistance of the electrodes is characterized by employing a semicircular diameter in the Nyquist designs.

In Fig. 3b, it can be observed that the resistance to charge transfer for the modified CoMnFeO_4_/FTO electrode is less than that of the bare electrode. It undoubtedly shows that the CoMnFeO_4_ nanoparticles act as a stimulus and speed up the interfacial charge transfer. The diameter of the semicircle in the curve corresponding to the ZnMnFeO_4_/FTO electrode is decreased in proportion to the CoMnFeO_4_/FTO electrode, which represents the high electrical conductivity of the ZnMnFeO_4_ compared to the CoMnFeO_4_ NPs. In the curve related to ZnMnFeO_4_/CoMnFeO_4_/FTO, a decrease in charge transfer resistance can be seen after coating the ZnMnFeO_4_ on the CoMnFeO_4_/FTO, which is a result of a higher electrochemical active surface area and the enhanced charge transfer rate of ZnMnFeO_4_/CoMnFeO_4_/FTO compared to the other modified electrodes. The Warburg element is also seen in the impedance spectrum (Fig. 3b), indicating the electrolyte diffusion into the coating and showing the porosity of the modifier. This is an essential and critical parameter in the properties of catalysts. It indicates that these two NPs together are being used successfully and have a positive effect on each other.

The electrochemically active surface area of the modified electrode was studied, and results were reported in the supplementary file (Fig. S1).

### Electrochemical behavior of hydrazine on ZnMnFeO_4_/CoMnFeO_4_/FTO

**Figure 4:**
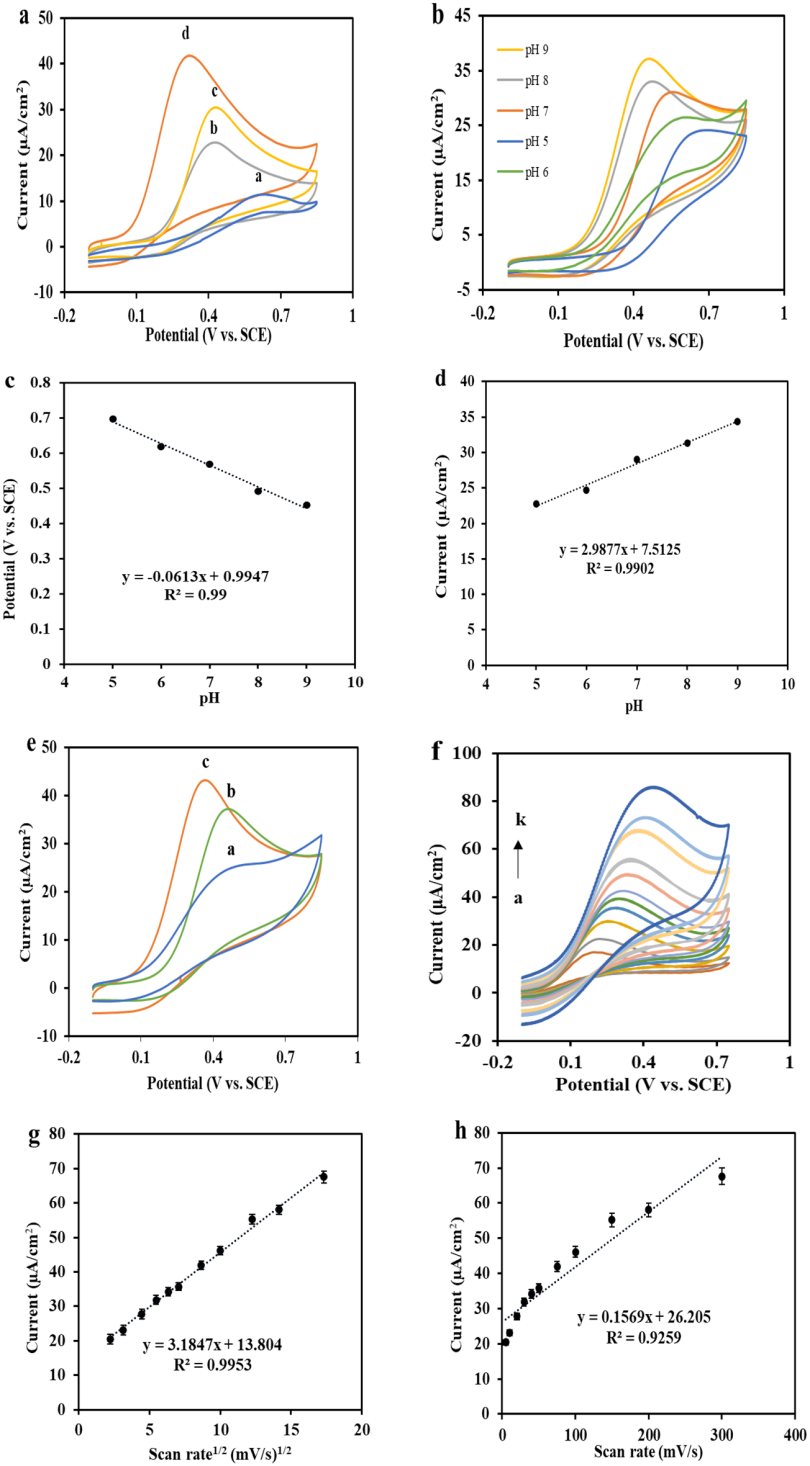

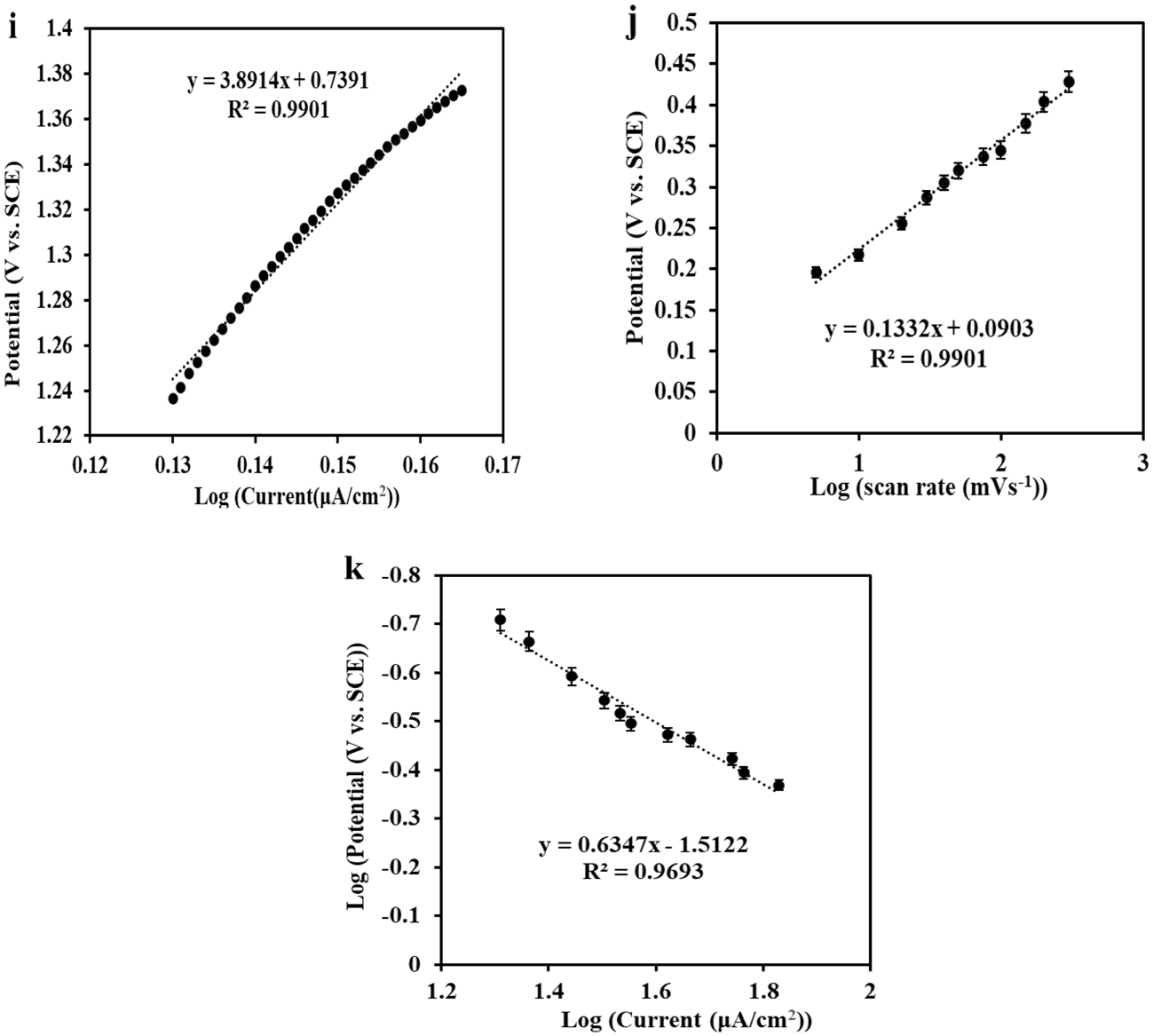
(**a**) CV responses of 100 µM hydrazine in 0.1 M ammonia buffer with pH = 9 for (a) Bare FTO (b) CoMnFeO_4_/FTO (c) ZnMnFeO_4_/FTO (d) ZnMnFeO_4_/CoMnFeO_4_/FTO at the scan rate of 50 mV s^−1^. (**b**) CV responses of ZnMnFeO_4_/CoMnFeO_4_/FTO electrode in phosphate buffer (0.1 M) containing 100 µM hydrazine at various pH from 5.0 to 9.0 (right to left) with the scan rate of 50 mV s^−1^, (**c**) Peak potential plot versus pH values, (**d**) Peak current plot versus pH values (**e**) checking out the buffer type; (a) Britton–Robinson, (b) Phosphate buffer, (c) Ammonia buffer. (**f**) CV responses of ZnMnFeO_4_/CoMnFeO_4_/FTO electrode in 0.1 M ammonia buffer (pH = 9.0) containing 100 µM hydrazine at various scan rates from 5 to 300 mV s^−1^ (from a to k). Inset: (**g**) and (**h**) diagrams of peak current vs. the square root of scan rates and scan rates, respectively. (**i**) Logarithm of peak current plot against peak potential, (**j**) Logarithm of peak current against logarithm of scan rates, (**k**) Logarithm of peak potential vs. logarithm of peak current.

The cyclic voltammetry technique was conducted to assess the electrochemical behavior of hydrazine on manifold-modified electrodes. The cyclic voltammetric response of 0.1 mM hydrazine in the 0.1 M ammonia buffer (pH = 9.0) was recorded in the potential range of − 0.1 to 0.85 V. As reflected in Fig. 4a, the oxidation of hydrazine on a bare FTO substrate demands a high positive potential (0.575 V), which shows a considerably lower peak current (curve a). In the b-curve, the hydrazine oxidation is shifted towards a less positive potential (0.423 V) by modifying the electrode surface with CoMnFeO_4_ nanoparticles, and the current is increased relative to the bare electrode state (22.0 μA). On the other side, in the c-curve that corresponds to the ZnMnFeO_4_/FTO electrode, the hydrazine oxidation potential is moderately shifted towards the positive potential (0.431 V) in comparison with the CoMnFeO_4_/FTO electrode, but the current is increased to about 29.0 μA. It does indicate that the electrodes modified with CoMnFeO_4_ NPs and ZnMnFeO_4_ NPs have catalytic properties on hydrazine. In the d-curve, it can be seen that when the electrode is modified with ZnMnFeO_4_/CoMnFeO_4_ NPs, there is a shift towards less positive potentials in the oxidation potential of hydrazine and also a sharp peak at potentials less than 0.4 V, about 0.34 V, and the current is increased to about 39.0 μA.

Given that, the ZnMnFeO_4_/CoMnFeO_4_/FTO has a better electrocatalytic property for hydrazine when compared with the other modified electrodes. A stable current was achieved in less than 5 s by adopting a modified electrode. It indicates a rapid electron exchange on the modified electrode surface and satisfactory catalytic performance.

### Effects of buffer type and pH value

To verify the effect of the electrolyte solution pH value, the 0.1 M phosphate buffer was used in the pH range of 5.0–9.0, including 0.1 mM hydrazine, reaching the greatest current response and the best oxidation potential of the sensor for hydrazine. In Fig. 4b, it is apparent that the peak potential and peak current of the ZnMnFeO_4_/CoMnFeO_4_/FTO electrode are highly dependent on the pH of the solution. This is due to the shift of hydrazine oxidation peak potentials to negative potentials with increasing pH of the solution, based on the following Eq. (1):

1 $${\text{E}}^{0\prime } \;\left( {\text{V}} \right) \, = \, - 0.0613\;{\text{ pH }} + \, 0.9947$$

The slope of − 61.3 mV was obtained from the potential of the E_p_–pH diagram, which is close to the theoretical Nernst value (Fig. 4c). Accordingly, it indicates that equal numbers of electrons and protons were engaged in the oxidation reaction of hydrazine (Eq. 2).

2 $${\text{N}}_{2} {\text{H}}_{4 \, } \to {\text{N}}_{2} + \, 4{\text{e}}^{ - } + \, 4{\text{H}}^{ + }$$

The pH value of the supporting electrolyte is a significant parameter for the effective electrocatalytic behavior of hydrazine, and as shown in Fig. 4d, the response of hydrazine increased along with an increasing pH value. It has been suggested that the enhancement of the current response in alkaline solutions is caused by the adsorption of hydrazine to the electrode surface. In this study, pH = 9.0 was chosen as the eligible pH, and indeed, the effect of buffer type on the electrooxidation of hydrazine was studied at the same pH value. Consequently, diverse buffers, namely ammonia, phosphate, and Britton–Robinson with a concentration of 0.1 M, were used to accomplish this. Figure 4e shows that the voltammetric response of the ammonia buffer is superior to that of buffers. The reason is that the hydrazine oxidation peak emerged at the lower potential in contradiction to other buffers and increased the current.

### Effect of scan rate

To investigate the kinetic reaction of the hydrazine oxidation and its electron transfer mechanism, the cyclic voltammetry technique was used at 5–300 mV s^−1^ scan rates by ZnMnFeO_4_/CoMnFeO_4_/FTO electrodes in 0.1 M ammonia buffer (pH 9.0) containing 0.1 mM hydrazine (Fig. 4f). According to the results, the peak current increases progressively as the scan rate increases. The diagrams of peak currents (I_p_) versus scan rate (ν) and second root of scan rate (ν 1/2) were plotted to perceive and interpret the diffusion or absorption nature of the electrode process. Considering that the peak currents are linearly proportional to the square root of the scan rate, the electrocatalytic oxidation of hydrazine on the ZnMnFeO_4_/CoMnFeO_4_/FTO was controlled by a diffusion-controlled process (Fig. 4g,h). As the scan rate increases, the peak potential of hydrazine electrooxidation shifts towards a positive potential. In other words, there are kinetic constraints at high scan rates^41^.

A Tafel plot was depicted at the scan rate of 5 mV s^−1^ (Fig. 4i) for further studying the kinetic parameters (α). According to Tafel Eq. (3):

3 $$\log i = \, \log i_{0} + \, (1 \, - \alpha )n_{a} F/ \, 2.303\;RT$$

where α is the transfer coefficient, T is the temperature (K), F is the Faraday constant (96,485 C mol^−1^), R is the universal gas constant (8.314 J K^−1^ mol^−1^) and *n*_*a*_ refers to the number of transferred electrons in determining step, The Tafel plot slope is 6.2156 mV decade^−1^. On this account, by substituting these values in Eq. (3), the α parameter is obtained at 0.77 assuming *n*_*a*_ equals 1. The relationship between peak potential (E_p_) and the natural logarithm of scan rate (log ν) can be defined by the Laviron equation.

According to this equation and also by using the slope value of the E_pa_ vs. log ν diagram (Fig. 4j) and the α value that was attained to be 0.77 from Eq. (3), the number of electrons engaged in the rate-limiting step of hydrazine (n) was estimated to be 1.

Additionally, a linear relationship between the E_pa_ and I_pa_ was proposed by depicting the diagram of log E_pa_ versus log I_pa_ (Fig. 4k) in the scan rates work. Accordingly, the regression equation was: log E_pa_ (V) = 0.6347 log I_pa_ (μA) − 1.5122 (R^2^ = 0.9693). The hydrazine oxidation, however, becomes more challenging in high-rate scanning because, by increasing the scan rate, a considerable change occurs with expanding peak currents in the hydrazine oxidation potential displacement towards more anodic potentials.

Based on all previous findings and results, the hydrazine electrochemical oxidation mechanism has been reported to be conducted via a 4-electron process and results in the production of nitrogen gasses by the following equations (Eq. 6)^18,42^.

The present survey found that the hydrazine oxidation process is an irreversible oxidation process in which hydrazine oxidizes to produce N_2_ and hydronium ions (H_3_O^+^) along with four electrons. In this part, the rate-limiting step is the first step containing one-electron transfer (Eq. 4), followed by the fast second step containing the three-electron transfer process (Eq. 5)^34,42^:

4 $${\text{N}}_{2} {\text{H}}_{4} + {\text{ H}}_{2} {\text{O}} \rightleftharpoons {\text{N}}_{2} {\text{H}}_{3} + {\text{ H}}_{3} {\text{O}}^{ + } + {\text{ e}}^{ - } \; \left( {{\text{rate - determining}}\;{\text{step}}} \right)$$

5 $${\text{N}}_{2} {\text{H}}_{3} + \, 3{\text{H}}_{2} {\text{O}} \rightleftharpoons {\text{N}}_{2} + \, 3{\text{H}}_{3} {\text{O}}^{ + } + \, 3{\text{e}}^{ - } \; \left( {{\text{fast}}\,{\text{step}}} \right)$$

6 $${\text{N}}_{2} {\text{H}}_{4} + \, 4{\text{H}}_{2} {\text{O}} \rightleftharpoons {\text{N}}_{2} + \, 4{\text{H}}_{3} {\text{O}}^{ + } + \, 4{\text{e}}^{ - } \; \left( {{\text{overall}}\;{\text{ reaction}}} \right)$$

### Chronoamperometric studies

The chronoamperometry technique was performed in a pH 9.0 ammonia buffer (0.1 M) containing different concentrations of hydrazine (from 15 to 75 µmol L^−1^) to ascertain the diffusion coefficient of the hydrazine. The potential of the working electrode was set at 0.4 V, and the Cottrell equation was applied (Eq. 7) to define the current response for an electroactive compound that is controlled by a diffusion mechanism:

7 $${\text{I}} = {\text{nFAD}}^{1/2} {\text{C}}\pi^{ - 1/2} {\text{t}}^{ - 1/2}$$

**Figure 5:**
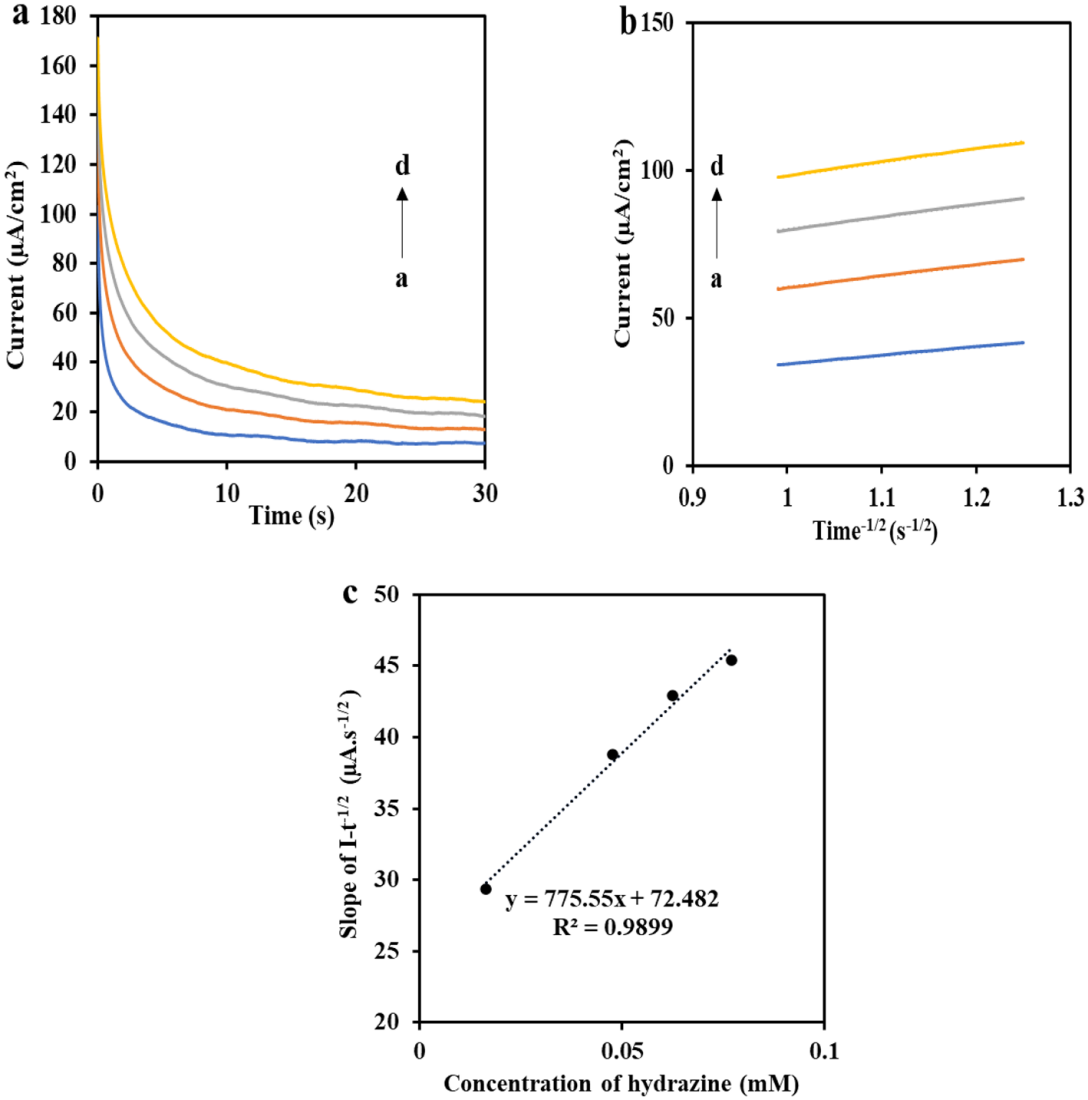
(**a**) Chronoamperograms achieved from ZnMnFeO_4_/CoMnFeO_4_/FTO electrode in the presence of 16, 48, 64 and 80 µM hydrazine (a–d) at the potential of 0.4 V vs. SCE, (**b**) Peak current of a, b, c and d curves vs. time^−1/2^ for varied concentrations of hydrazine (16–80 µM); and (**c**) is slope values of I–t^−1/2^ plot vs. various concentrations of hydrazine.

C and D are the bulk concentration (mol cm^−3^) and diffusion coefficient (cm^2^ s^−1^), respectively; A is the active area of the modified electrode that was obtained to be 1.72 cm^2^, and I refers to the diffusion current of hydrazine from the bulk solution to the interface of the solution/electrode, and the other signs have their specific conventional meanings. The experimental diagrams that have been plotted for the several hydrazine concentrations are viewed in Fig. 5a. The diagram of I versus t^−1/2^ can be observed in Fig. 5b. For hydrazine, the average diffusion coefficient was computed by plotting the slope values versus different hydrazine concentrations (Fig. 5c); D = 4.29 × 10^−6^ (cm^2^ s^−1^).

### Determination of hydrazine

To assign the detection limit and the linear range as the critical parameters of an electrochemical nano-sensor, the differential pulse voltammetry procedure was implemented with optimized parameters such as a scan rate of 50 mV s^−1^, pulse amplitude as 0.05 V, pulse time as 0.05 s, step potential as 0.005 V, quiet time as 3 s, and interval time as 0.5 s, and the DPV curves of ZnMnFeO_4_/CoMnFeO_4_/FTO were recorded with different concentrations of hydrazine in 0.1 M ammonia solution (pH 9.0).

**Figure 6:**
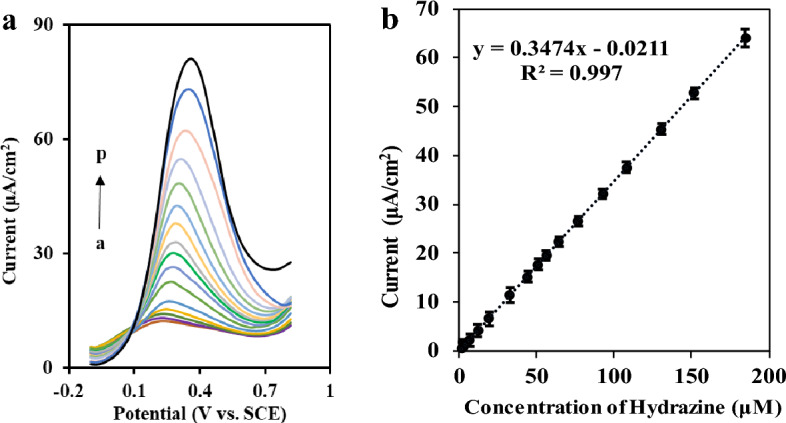
(**a**) Differential pulse voltammograms (DPV) for varied concentrations of hydrazine (a–p) in 0.1 M ammonia buffer with pH = 9.0 and (**b**) is the calibration plot of hydrazine with a linear range of 1.23–184.7 µM.

Figure 6a shows that as the concentration of hydrazine increases, the electrocatalytic response gets sharper. A linear calibration plot was also attained between the hydrazine concentrations and associated peak current (I_p_) as I (μA) = 0.347 [hydrazine] (μA/mM) + 0.021; R^2^ = 0.997. The linear range was achieved at the concentration range of 1.2–184.7 µM. Sensitivity, LOD, and LOQ are estimated as 0.347 μA mM^−1^, 0.82 μM and 2.75 μM for hydrazine, respectively, through the slope of the calibration plot (Fig. 6b).

ZnMnFeO_4_/CoMnFeO_4_ as a modifier provides an acceptable ambiance for hydrazine indication since it has a high electroactive surface area. It can be noted that the high electron communication features are caused by the high sensitivity of this sensor, which enhances the direct charge transfer between the active area of the modifier and the FTO substrate. The analytical performance of the suggested sensor that has been used to detect hydrazine compared to the electrodes reported earlier is shown in Table 1.

**Table 1:** The comparison of the analytical performance of the suggested sensor with other modified electrodes which have been used to detect hydrazine.

Electrode	Linear range (mM)	LOD (µM)	References
Co-doped CeO_2_/GCE	0.0072–1	7.2	^43^
PANI/g-C3N4/AgNPs/FTO	5–300	300	^16^
Zn-MOF/GCE	0.02–0.35	2	^44^
TiO_2_ Nanowires/FTO	0.01–1	1.91	^45^
Quinoline quinones@MWCNT/GCE	0.025–0.45	12	^46^
PdNPs-β-CD/rGO	0.00005–1.6	0.028	^47^
NiO NF/GCE	0.99 × 10^−3^–98.13 × 10^−3^	0.0898	^48^
RGO/Pt‐TPP	13 × 10^−6^–232 × 10^−3^	0.005	^49^
GCE/MWCNT@Ind-Oxid	10 × 10^−3^–5	0.22	^50^
Zn-MOF@rGO/AuE	1 × 10^−9^–1 × 10^−6^	8.7 × 10^−3^	^19^
Glucose derived sheet-like carbons-NiO	0.0005–12	1.5	^51^
ZnO Nanorods/FTO	0.0003–3	515.7	^21^
NiHCF/CCE	0.02–2	8	^52^
ZnMnFeO_4_/CoMnFeO_4_/FTO	0.0012–0.1847	0.82	This work

The detection limit of the suggested sensor is better or at least similar to other modifiers of electrodes that have been previously stated in the table. The findings demonstrate that the sensor was adequate and qualified for hydrazine detection.

### Sensor selectivity

The concentrations of some anions and cations and several chemicals, including Cl^−^, Br^−^, NO_3_^−^, SO_4_^2−^, (CH_3_CO_2_)^−^, Na^+^, K^+^, Cu^2+^, Ni^2+^, ethanol, citric acid, uric acid, glucose (GL), Lactose, Fructose, and ascorbic acid (AA), were added separately to the ammonia buffer solution (0.1 M, pH 9.0) containing 100 µM hydrazine. This was done to evaluate the selectivity of the ZnMnFeO_4_/CoMnFeO_4_/FTO and examine the absence of interference, which is an influential and essential parameter in practical applications. For the maximum concentration of the foreign substances, the limit of tolerance was taken, which produced an approximate error in the analytical response of the analyte of less than ± 5% (Fig. S2). As can be observed in Table 2, the small change in the DPV response of hydrazine is caused by the eightfold ascorbic acid and uric acid, tenfold citric acid, 300-fold glucose, Lactose and Fructose, and 500-fold ethanol, K^+^, Na^+^, Cu^2+^, Ni^2+^, Br^−^, Cl^−^, SO_4_^2−^, (CH_3_CO_2_)^−^, and NO_3_^−^. According to the findings, the suggested sensor has been chosen properly. Therefore, it can be applied to detect hydrazine in the presence of biological molecules and environmental pollutants.

**Table 2:** Tolerance limit of interferences on DPV response of hydrazine.

Substance	Molar ratio of substance to hydrazine	Bias %
ETOH	500	+ 0.41
KBr	500	+ 1.22
NaCl	500	+ 0.82
NaNO_3_	500	− 1.63
Ni(CH_3_CO_2_)_2_	500	− 3.26
CuSO_4_	500	− 2.04
Glucose	300	+ 3.67
Lactose	300	+ 2.86
Fructose	300	+ 1.22
Citric acid	10	− 0.82
Uric acid	8	+ 4.49
Ascorbic acid	8	− 4.88

### Real samples

For real samples of hydrazine, selective detection was provided by the suggested sensor in the present study, and it has been shown whether this method is applicable. The hydrazine and derivatives that concurrently exist in tobacco products. To assess the amount of hydrazine, however, the diluted tobacco solution was injected directly into the electrolyte solution. Next, the amount of hydrazine standard solution that had been assessed earlier was added, and then, to detect the concentration of hydrazine, the standard addition method by differential pulse voltammetry (DPV) technique was accurately implemented. In Fig. S3, it can be observed the test of common hydrazine in real samples for three brands of cigarettes (C_1_–C_3_) using ZnMnFeO_4_/CoMnFeO_4_/FTO. Considering that the findings of the calculated recovery were in the range of 97.77–100.71% with a R.S.D. (for three repetitions) of below 3%, one can deduce that the suggested sensor has a desirable selectivity (Table 3).

**Table 3:** Recovery test of hydrazine in real samples using ZnMnFeO_4_/CoMnFeO_4_/FTO.

Parameters	Cigarette #1 C1	Cigarette #2 C2	Cigarette #3 C3
Tobacco weight (gr)	4.2416	1.0903	2.8355
Diluted volume	50.0	50.0	50.0
Linear equation	y = 0.3444x + 1.1841	y = 0.4168x + 0.5617	y = 0.3641x + 1.6249
R^2^	0.9975	0.9931	0.9978
Detected value (µM)	3.45	1.35	4.46
R.S.D. detected value	2.82%	1.75%	1.07%
Spike	19.6	19.6	19.6
After spike (µM)	22.57	20.54	24.4
R.S.D. Spike	1.67%	1.91%	1.79%
Recovery	97.93%	97.98%	101%
Hydrazine per cigarette (ng)	551	216	715

### The stability, repeatability, and reproducibility of ZnMnFeO_4_/CoMnFeO_4_/FTO

The electrode was kept in the air for one month to analyze the modified electrode’s stability. Afterward, required assessments were conducted on the 7th and 30th days. Based on the findings of this study, it can be declared that the modified electrode has 97.25% of its initial current response after 7 days and 95.88% for a month, which has desirable stability in view of its high specific surface area (Fig. S4a). Employing five consecutive tests with the same modified electrode and applying cyclic voltammetry, the modified electrode response repeatability in hydrazine measurement was precisely measured. The R.S.D. of peak currents was estimated at 1.78%, which demonstrates satisfactory and adequate repeatability for the modified electrode (Fig. S4b). In addition, four different electrodes were modified with ZnMnFeO_4_/CoMnFeO_4_ for studying the reproducibility of the sensor, the CVs were registered, and the R.S.D. was calculated at 2.05% (Fig. S4c). The findings conclusively reveal that ZnMnFeO_4_/CoMnFeO_4_/FTO has good stability as a sensor and also possesses adequate quality of repeatability and reproducibility in hydrazine measurement.

The sensor's low reusability is one of the work's limitations. After four washes, the modified electrode's CV response recovered as much as 98.4%, 94.6%, 91%, and 82.9% of the original response signal (Fig. S4d).

## Conclusion

In this study, a new assay for the oxidation of hydrazine was prepared based on the modified FTO electrode. The modified FTO electrode was prepared as ZnMnFeO_4_/CoMnFeO_4_/FTO through deposition of the ZnMnFeO_4_ NPs on the surface of the CoMnFeO_4_ NPs. The results showed that this modified electrode provides significantly enhanced electrolytic activity with a remarkable decrease in overvoltage and provides better peak current intensity when compared with bare FTO electrodes. To detect hydrazine using the DPV technique, the modified electrode showed high sensitivity and selectivity and a low detection limit. Furthermore, there are several remarkable advantages to the suggested sensor, including long-term stability and repeatability, simple preparation, and low cost.

The interference from current co-existing chemical species, which are present in excess concentration, was tolerated by the examined electrode. In the final analysis, the modified electrode was employed to detect the amount of hydrazine in cigarette samples, and the findings were satisfactory.

## Supplementary Information

Supplementary Information.

## Data Availability

The datasets generated during the current study are available from the corresponding author on reasonable request.

## Abbreviations

DPV: Differential pulse voltammetry
Eq: Equation
FTO: Fluorine doped tin oxide
Fig: Figure
LOD: Limit of detection
LOQ: Limit of quantification
RSD: Relative standard deviation
NPs: Nanoparticles
