# Supplementary material for: Enhanced electrocatalytic activity of fluorine doped tin oxide (FTO) by trimetallic spinel ZnMnFeO4/CoMnFeO4 nanoparticles as a hydrazine electrochemical sensor
Source: Sci Rep. 2023 Jul 27;13:12188. doi: 10.1038/s41598-023-39321-0 (PMC10374622; doi:10.1038/s41598-023-39321-0)
Supplement: Supplementary file 1 — Supplementary Information. [file 41598_2023_39321_MOESM1_ESM.docx]

**Supplementary Material File**

Enhanced electrocatalytic activity of Fluorine Doped Tin Oxide (FTO) by Trimetallic spinel ZnMnFeO_4_/CoMnFeO_4_ nanoparticles as a hydrazine electrochemical sensor

*Jalal Niazi Saei^1^, Karim Asadpour-Zeynali^1,2^[[1]](#footnote-1)^*^*

*^1^Department of Analytical Chemistry, Faculty of Chemistry, University of Tabriz, Tabriz 51666-16471, Iran*

*^2^Pharmaceutical Analysis Research Center, Faculty of Pharmacy, Tabriz University of Medical Sciences, Tabriz 51664, Iran*

- 1. **Apparatus and software**

All electrochemical experiments were conducted without eliminating dissolved O_2_ at room temperature. In order to measure the pH, a digital pH meter (model Metrohm 827 pH meter) was employed. A three-electrode system has been applied for electrochemical measurements containing modified fluorine-doped tin oxide (FTO) with ZnMnFeO4/CoMnFeO4 nanoparticles (FTO) as the working electrode. A FTO glass substrate has been bought from Nano Gostar Company of Iran, Isfahan. Saturated calomel (SCE) as the reference electrode, and as an auxiliary electrode, a Pt wire that is immersed directly in solution has been used, respectively (SCE and Pt wire electrodes purchased from Azar Electrode Company (Urmia, Iran)). All potentials are reported relative to the reference electrode. Electrochemical measurements were carried out by employing a Metrohm 757 VA computer electrochemical workstation that has been equipped with a three-electrode cell.

With the aim of verifying of the associated bands' identification, the Fourier-transform infrared (FT-IR) spectra were recorded on a Bruker Optics TENSOR 27 spectrometer. The structure of the crystalline phase and the purity of the spinel oxide phase were discerned by making use of the X-ray diffraction (XRD) technique; their patterns were recorded with the Phillips PW 1730 instrument (Eindhoven, Netherlands) with Cu-Kα radiation (λ = 0.154056 nm). Field emission scanning electron microscopy (FESEM) equipped with energy-dispersive X-ray spectroscopy (EDX) was enforced on the Tescan Mira 3 FESEM with the gold coating system to examine the morphological observation and elemental composition of the developed and prepared sample. Moreover, the transmission electron microscopy (TEM) method was applied to the Zeiss EM-10C microscope (Germany) at an accelerating voltage of 100 kV to analyze the particle size and textural features. Besides, an ultrasonic bath (FALC, Italy) was utilized to clean the electrode surface.

- 1. **Reagents and solutions**

Hydrazine (HZ), iron (III) nitrate nonahydrate (Fe(NO_3_)_3_.9H_2_O), manganese dinitrate tetrahydrate (Mn(NO_3_)_2_.4H2O), zinc dinitrate tetrahydrate (Zn(NO_3_)_2_.4H_2_O), cobalt dinitrate hexahydrate (Co(NO_3_)_2_.6H2O), and ammonia solution (25% v/v) were procured from Merck Company (Germany). Ammonia, phosphate, and Britton-Robinson buffers with a concentration of 0.1 M have been utilized. In order to prepare all of the solutions, deionized water was used. 0.1 M ammonia buffer (pH 9.0) was used as a carrier electrolyte for HZ. All compounds were used in the absence of any further purification.

- 1. **Electrochemical *measurements***

In accordance with the conditions of the device, the calibration curves for HZ have been schemed using the DPV technique. The parameters, including step potential = 0.005 V, modulation time = 0.05 s, modulation amplitude = 0.025 V, quiet time = 5 s, and interval time = 0.5 s, are the optimized DPV parameters.

Aiming to indicate the application of a modified electrode, the suggested sensor has been evaluated with no pretreatment by using cigarettes, which were the real sample for HZ in this study. These cigarettes were purchased from a local supermarket and were chosen from three famous brands. In the first place, the tobacco content from the cigarette was removed in order to prepare the real sample. The sample was initially weighed and then leached in 0.1 M, pH 9.0 ammonia buffer, and after all that, ultrasonication of the sample was done, which took 10 min. Finally, for the purpose of analyzing the real sample, the filtrated solution was diluted properly. In 5 mM [Fe(CN)_6_]^-3/-4^ containing 0.1 M KCl, all measurements of electrochemical impedance spectroscopy were carried out accurately. The amplitude of the applied sine wave potential was 5 mV. The total EIS measurements were recorded and registered at open circuit potential (OCP) in the frequency range of 100 kHz to 0.01 Hz.

- 1. **Study of the active surface area of the HZ sensor**

In order to check the electrocatalytic activity of a modified electrode, it is extremely important to recognize the real active surface area of that electrode. For calculating the microscopic surface area of the ZnMnFeO_4_/CoMnFeO_4_/FTO electrode, the cyclic voltammetric method was applied in a 0.1 M KCl solution containing 5 mM of [Fe(CN)_6_]^-3/-4^ as a redox probe at different scan rates, which can be seen in Fig. S1a, and the record of I_p_-ν^1/2^ diagram is presented in Fig. S1b.

The electrochemical active surface area of an electrode was estimated from the slope value of the peak current diagram’s opposing scan rates in the Randall-Sevcik equation [1]: I_p_ = (2.69 × 10^5^) n^3/2^ AD^1/2^ ʋ^1/2^ C (1). In this equation, I_p_ refers to the peak current (A), C is the concentration [Fe(CN)_6_]^-3/-4^ (mol.cm^-3^), υ is scan rate (V.s^-1^), the number of electrons exchanged is presented with n, D refers to the diffusion coefficient (cm^2^.s^-1^) and A refers to the microscopic surface area of the electrode (cm^2^). In the KCl solution, the coefficient of diffusion for the required probe is 0.2 M equal to 7.6 × 10^-6^ (cm^2^.s^-1^).

The effective electrochemical surface area was computed at 1.72 cm^2^ for ZnMnFeO_4_/CoMnFeO_4_ NPs. This implies the fact that the modifier has enhanced the effective electrochemical surface of the FTO glass substrate and qualified this sensor for electrochemical sensing.

| 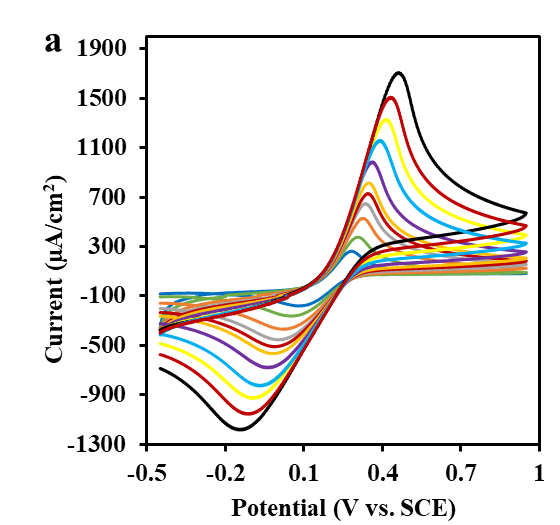 | 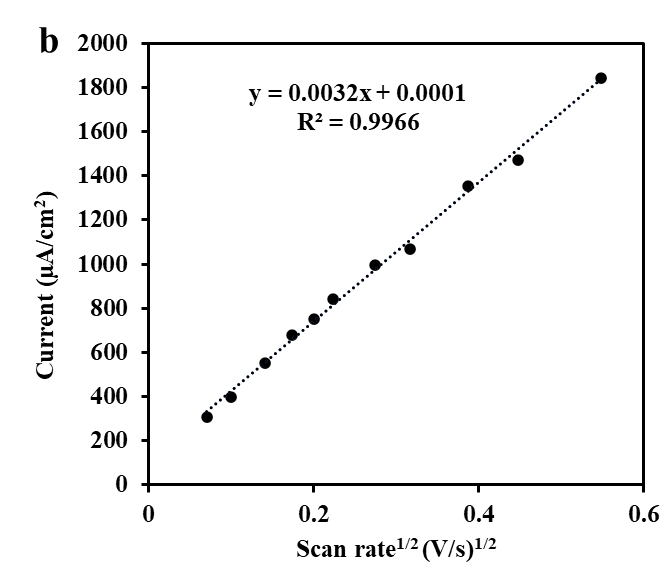 |
| --- | --- |

**
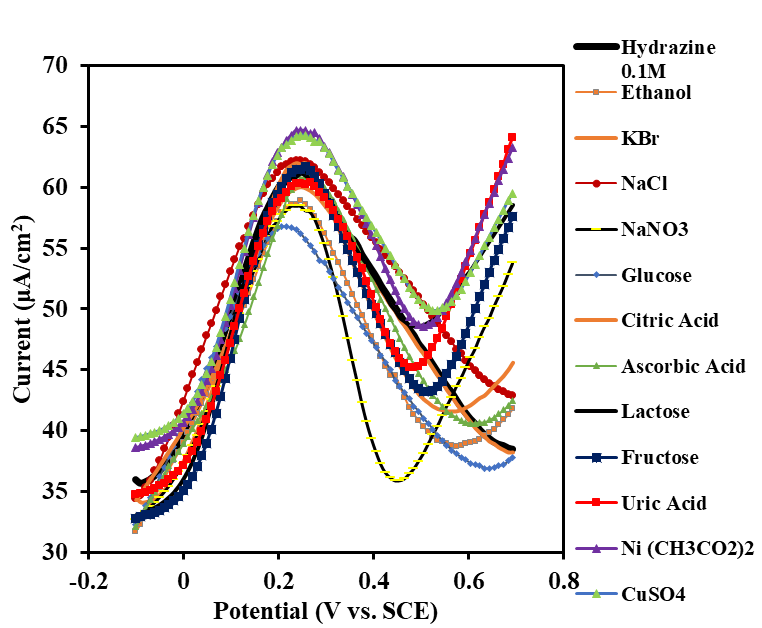
Fig. S1.** **(a)** CV responses of the ZnMnFeO_4_/CoMnFeO_4_/FTO in 5mM [Fe(CN)_6_]^-3^/[Fe(CN)_6_]^-4^ containing 0.1 M KCl at the scan rates of 5, 10, 20, 30, 40, 50, 75, 100, 150, 200, 300 mV.s^-1^ (inner to outer), **(b)** Diagram of peak current against square root of the scan rates.

| **Fig. S2**. Differential pulse voltammograms (DPV) for 100µM hydrazine in 0.1 M ammonia buffer with pH = 9 in absence and presence of interferences. | |
| --- | --- |
| 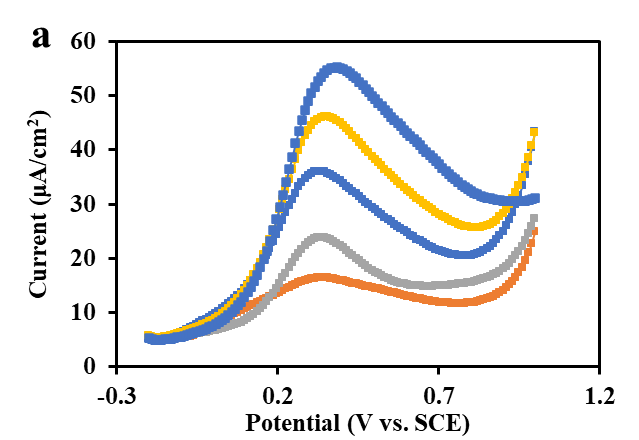  **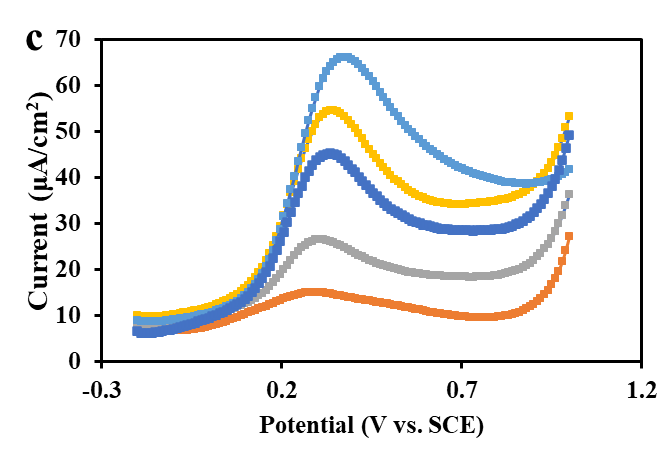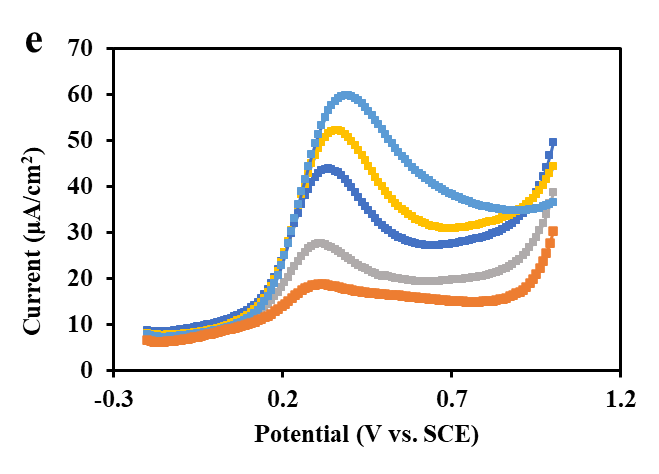** | 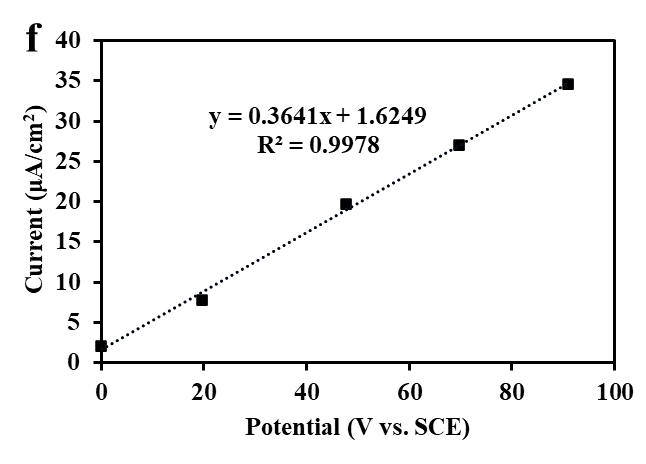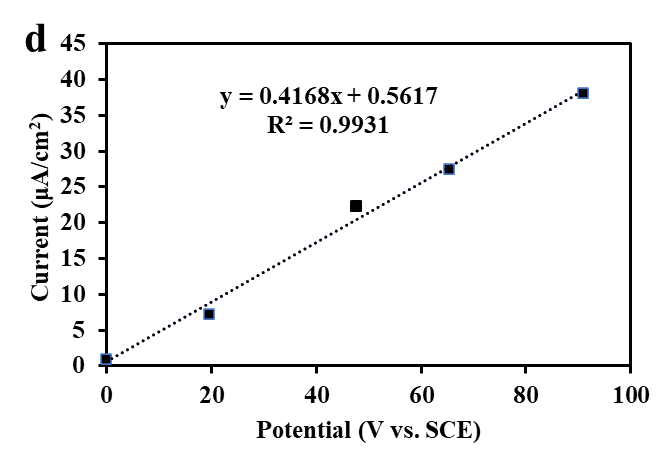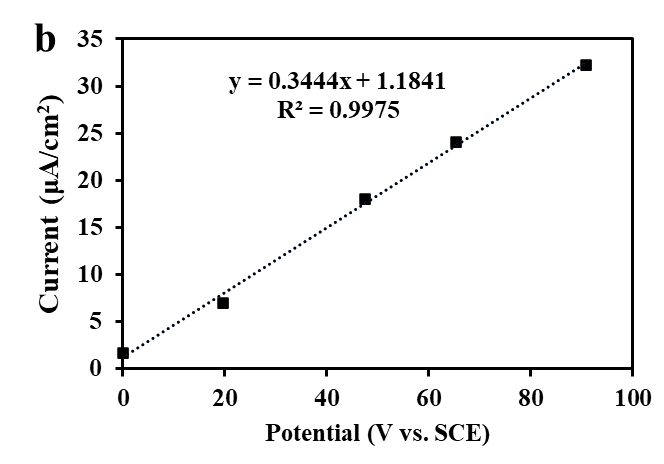 |

**Fig. S3.** (**a, c, e)** DPV and **(b, d, f)** are the calibration plot of the unknown sample and spiked concentrations of 19.6, 47.6, 65.42 and 90.91 µM of hydrazine for the Cigarette #1, #2 and #3 respectively.

| 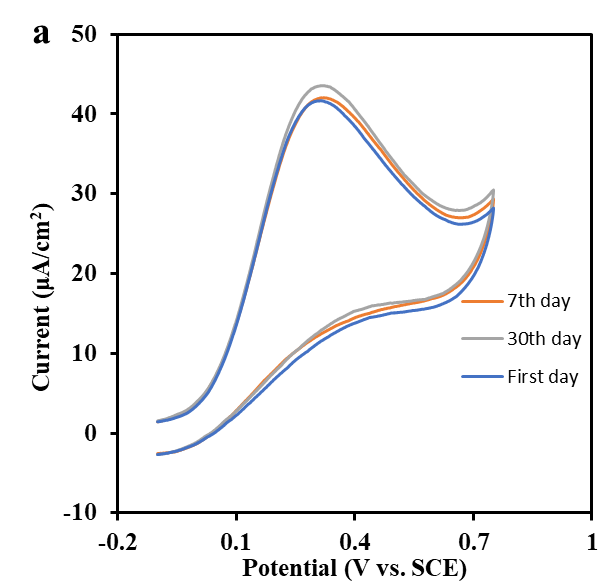 | 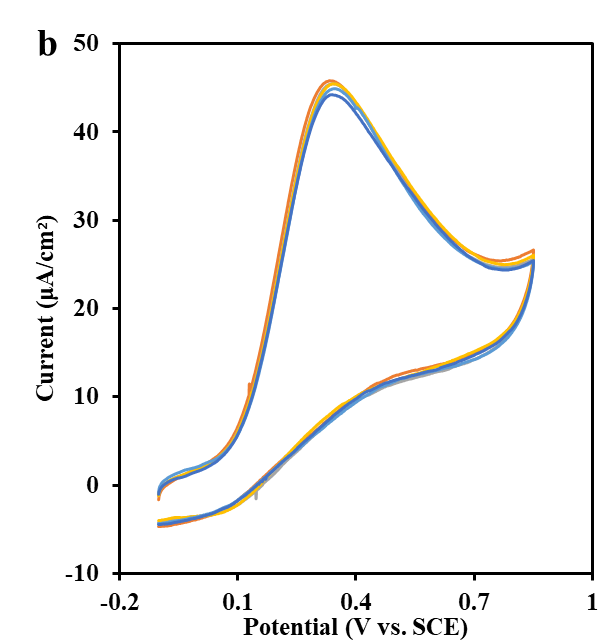 |  |
| --- | --- | --- |
| 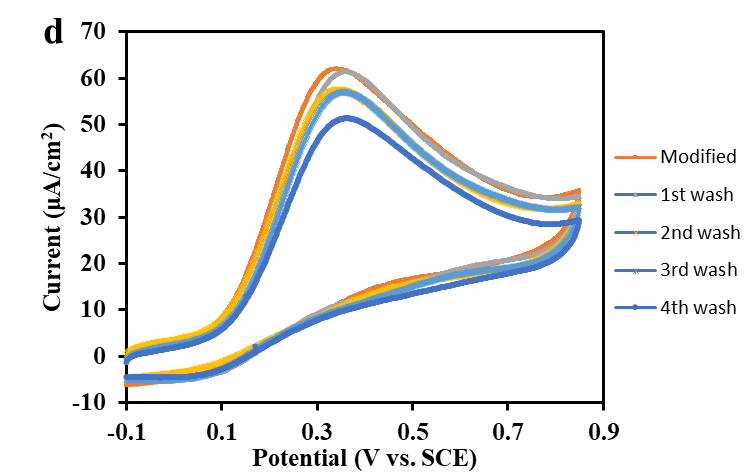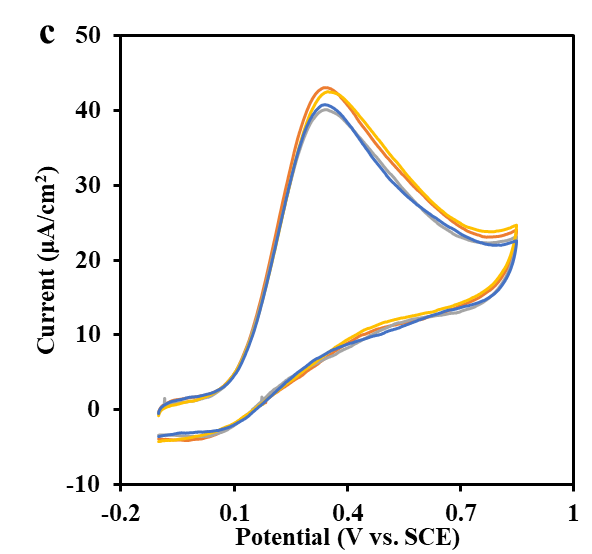 | | |

**Fig. S4.** **(a)** stability of the ZnMnFeO_4_/CoMnFeO­_4_/FTO electrode at the first, 7^th^ and 30^th^ days; **(b)** is repeatability, **(c)** is the reproducibility and **(d)** is the reusability of the ZnMnFeO_4_/CoMnFeO­_4_/FTO electrode after washed for 4 times.

1. * Corresponding author: K. Asadpour-Zeynali

   Tel.: +98 413 3393113; Fax: +98 413 3340191.

   E-mail address: [asadpour@tabrizu.ac.ir](mailto:asadpour@tabrizu.ac.ir); [k.zeynali@gmail.com](mailto:k.zeynali@gmail.com) [↑](#footnote-ref-1)
